# Exploring Developmental Connections: Sleep Patterns, Self‐Locomotion, and Vocabulary Growth in Early Childhood

**DOI:** 10.1111/infa.12650

**Published:** 2025-01-24

**Authors:** Margherita Belia, Marilyn Vihman, Tamar Keren‐Portnoy

**Affiliations:** ^1^ Language and Linguistic Science Department University of York York UK

**Keywords:** crawling, dynamic systems, sleep, vocabulary growth, walking

## Abstract

Current research indicates likely developmental connections between the evolution of sleep patterns, motor skills progression, and the expansion of vocabulary. These connections are grounded in the well‐established role of sleep in memory and learning, as well as in the cascading effects on language development of the acquisition of new motor skills. However, no study has so far undertaken a comprehensive and systematic examination of these connections or explored their developmental trajectory over time. Yet understanding vocabulary development depends on considering development in the sleep regulation and motor domains, to provide a biologically grounded explanation of how early lexicons are built and strengthened. This study investigates the links between vocabulary growth and two significant changes occurring over the first 2 years of life: self‐initiated locomotion and the consolidation of overnight sleep. Our results reveal mutual associations between these domains, which tend to emerge during periods of marked developmental change in language, motor skills, and sleep patterns regulation. Moreover, these associations were observed to change over time, suggesting dynamic interconnections between these developmental domains. Our findings point toward the importance of investigating vocabulary development from a dynamic systems perspective, as the product of continuous interactions between cognition, the body and the environment.

## 1 Background

Infancy is a period of outstanding plasticity, where small changes in one domain can significantly influence changes in others (Karmiloff‐Smith 1998). Studying domains in isolation risks missing crucial associations and “decontextualizing the child” (Scholnick 2001). From a biological perspective development is best viewed holistically, at both individual and global timescales (Thelen and Smith 1994).

Cognition and physiology are inseparably linked in development: Cognition is rooted in physiology, as it is embodied in a growing, developing organism. Therefore, language development should be studied within the context of an evolving body that also changes how it moves and interacts with the environment (Iverson and Thelen 1999; Iverson 2010). Additionally, language acquisition should be considered in relation to the biological processes that underlie learning, memory and cognitive function—processes in which sleep plays a key role (see Rasch and Born 2013). Therefore, sleep patterns, which change dramatically as infants grow (Galland et al. 2012; Wielek et al. 2019), should have complex effects on language and other areas of development.

While previous research has found links between sleep and language development, sleep and motor development, and motor and language development, no study to our knowledge has systematically examined the relationships among these three domains over time. Our study aims to fill this gap by exploring whether and how sleep patterns, self‐initiated locomotion, and vocabulary development interact throughout the first 2 years of life. By examining these three domains together, we seek to provide new insights on how vocabulary develops, offering a more complete picture than can be afforded by studies that focus on only one or two of these domains at a time. This integrated approach is rarely used in developmental science, where domains are often studied in isolation. However, we believe it is important because it can offer insight into the internal processes that play a role in vocabulary growth. Our study does not test specific hypotheses; instead, it aims to explore these associations and lay the groundwork for future research.

It is important to note that most research on sleep pattern development has been conducted with populations in industrialized settings and specific geographical regions, which may not capture the full diversity of sleep habits observed worldwide. Factors such as access to artificial light, seasonal variations, and culturally specific caregiving practices (e.g., co‐sleeping, scheduled sleep times) all influence the development of sleep patterns in different contexts (Owens 2004; Jeon, Dimitriou, and Halstead 2021). Similarly, the onset of motor milestones, such as crawling and walking, is influenced by cultural practices and caregiving environments. Specific caregiving practices can either accelerate or delay these milestones by several months (Adolph and Hoch 2019; Holden et al. 2022). These cultural differences not only affect motor skill development but also impact the opportunities for communicative development. Our study focuses on a population of infants in the UK, primarily from White British/Irish families with a homogeneous sociodemographic background of mid‐to‐high socioeconomic status. Consequently, the literature we review and our findings are based on this population and may not be directly applicable to other, more diverse groups.

After an overview of the associations between sleep patterns in infancy and their relationship to development in general, we will present the existing evidence on the associations between sleep and vocabulary development, vocabulary and the development of self‐locomotion and sleep and self‐locomotion. We will then present the study objectives in more detail.

### 1.1 Sleep Regulation and Development

Sleep development is a “bio‐psycho‐social process” (Paavonen et al. 2020, 152) influenced by a range of factors, including social and cultural contexts, the development of self‐regulatory mechanisms and wake‐sleep rhythms as well as neuroanatomical changes (France and Blampied 1999; Ednick et al. 2009). Therefore, sleep patterns exhibit considerable variability, both between and within individuals (Mindell et al. 2010; Tham, Schneider, and Broekman 2017). Nevertheless, a commonly observed phenomenon is sleep regulation, that is, the gradual transition to longer bouts of uninterrupted sleep, primarily occurring at night (also known as “sleep consolidation,” e.g., Dionne et al. 2011). Besides cultural practices, this transition is facilitated by the child's growing ability to self‐soothe, but it is also underpinned by fundamental neurophysiological and anatomical changes. These changes, occurring around the first year of life, include the maturation of brain structures involved in circadian rhythm regulation, the establishment of adult‐like sleep cycles, and the synchronization of sleep spindles, which are known to contribute to brain maturation, memory and learning (Ednick et al. 2009; Paavonen et al. 2020; Rivkees 2003). Research shows that sleep consolidation is chiefly driven by a reduction in the number of night awakenings and a shift toward a larger proportion of sleep occurring at night than during the day. This results in a decrease in nap frequency and overall time spent asleep, while night‐time sleep duration remains relatively stable (Iglowstein et al. 2003; Bruni et al. 2014; Paavonen et al. 2020; Rivkees 2003). Importantly, this shift becomes particularly pronounced in the second year of life, with a decrease from two naps to one at around 15–18 months (Galland et al. 2012) and a sharp decrease in night awakenings (Paavonen et al. 2020).

Recent neuroanatomical findings support the link between sleep regulation and brain development (see Mason, Lokhandwala, et al. 2021, for a review). For instance, hippocampal subfield volume is significantly smaller in 4‐year‐olds who still habitually take naps compared to same‐age peers who no longer nap regularly (Riggins and Spencer 2020). Given the pivotal role of sleep and of the hippocampus in memory and learning (e.g., Born and Wilhelm 2012), this has led to the hypothesis that letting go of frequent naps may be a marker of brain maturation and larger memory capacity. This account is supported by evidence indicating that preschoolers who continue to take naps habitually derive greater benefits from a post‐encoding nap in spatial memory (Kurdziel, Duclos, and Spencer 2013). In further support of this account, research suggests that preschoolers who no longer habitually nap perform equally well on novel word retention whether they nap or not, while habitual nappers experience a decline in memory if a nap does not occur between encoding and testing (Esterline and Gómez 2021). Although this evidence mainly pertains to children older than 2 years, evidence for overnight sleep consolidation influencing cognition comes from younger populations as well.

For example, Pecora et al. (2022) reported that longer daytime sleep in 8‐month‐olds was associated with higher cognitive development scores. Actigraphy studies have shown that more fragmented sleep and greater motor activity during sleep in 10‐month‐olds are associated with slower general development, with sleep efficiency (i.e., the time spent asleep over total bedtime hours) correlating positively with developmental scores (Scher 2005). Contrary to Pecora et al. (2022), Gibson, Elder, and Gander (2012) found that higher proportions of nocturnal sleep relative to daytime sleep in 11–13‐month‐olds were positively associated with cognitive and motor development. Therefore, the impact of sleep regulation on development might vary over the first year of life.

Sleep patterns in the early months can predict cognitive outcomes at later ages. For example, Touchette et al. (2007) found that shorter sleep duration in early childhood was linked to poorer cognitive performance and vocabulary knowledge in later years. In Pisch, Wiesemann, and Karmiloff‐Smith (2019), infants (aged 4, 8, and 10 months) who developed mature working memory capacities earlier also spent more time asleep at night in the first months of life. Slow wave activity (SWA), a typical electrophysiological phenomenon observed in the sleeping brain, improves the brain's ability to encode new information during subsequent wakefulness (Van Der Werf et al. 2009; Antonenko et al. 2013). Thus, more uninterrupted sleep at night may ensure full SWA functioning; this would enhance the brain's ability to learn new information in the wake time that follows (Pisch, Wiesemann, and Karmiloff‐Smith 2019). Additionally, in Bernier et al. (2010), higher proportions of nighttime sleep at 12 and 18 months predicted more advanced executive functioning at 18 and 26 months, respectively.

These findings collectively highlight the importance of overnight sleep consolidation in the development of higher‐order mental functions, general motor development and physical wellbeing. They also suggest that frequent napping in infancy may reflect less developed memory networks. The next sections will review evidence on the associations between sleep, vocabulary and self‐locomotion development.

### 1.2 Sleep Regulation and Vocabulary Development

Associations between sleep regulation and vocabulary development have been widely observed, though the findings are mixed. Lam et al. (2011) found that increased napping time in preschoolers (aged 3–5) correlated negatively with receptive vocabulary, suggesting that longer night‐time sleep may reduce the need for daytime naps and enhance neurocognitive performance. Longitudinal studies highlight the complex relationship between sleep regulation and vocabulary growth over time. For example, Horváth and Plunkett (2016) found that more frequent daytime naps between 8 and 38 months were positively associated with receptive vocabulary and, to a lesser extent, with productive vocabulary 3–6 months later. In addition, fewer hours of nighttime sleep predicted faster productive vocabulary growth. In contrast, other studies emphasize the importance of night‐time sleep. For example, Dionne et al. (2011) found that a smaller proportion of nighttime sleep at 6 months predicted poorer language outcomes at 18 and 30 months: Children who experienced language delays at 60 months had slept less at night between 6 and 18 months. Similarly, Knowland et al. (2022) found that longer, less interrupted night‐time sleep between 6 and 36 months predicted larger receptive vocabularies at school entry.

These findings align with the well‐established role of sleep and naps in word learning (Axelsson, Williams, and Horst 2016; Belia, Keren‐Portnoy, and Vihman 2023). Infants and toddlers who nap shortly after exposure to novel words tend to remember them better than infants who stay awake during the same period (e.g., Horváth et al. 2015; Axelsson et al. 2018; Williams and Horst 2014). Similar findings have been observed as early as 6 months of age for word learning tasks like category learning (e.g., Friedrich et al. 2017) and the extraction of word forms from strings of nonwords in an artificial language (Gómez, Bootzin, and Nadel 2006; Simon et al. 2017). Napping shortly after learning also has long‐lasting effects on memory, with improved performance noted 24 h later (Werchan, Ji‐Soo, and Gómez 2021; Hupbach et al. 2009; Belia, Keren‐Portnoy, and Vihman, in press). The number and habituality of naps may play a mediating role. For example, Mason, Kurdziel, and Spencer (2021) found that two naps were optimal for consolidating newly learned information at 9 months. However, after the second birthday, longer and more frequent daytime naps were associated with shorter and more disrupted night sleep (Thorpe et al. 2015). Nevertheless, children up to 4 years old still benefited from daytime naps in consolidating spatial memories and novel words, especially if they were still taking naps habitually (Kurdziel, Duclos, and Spencer 2013; Esterline and Gómez 2021).

Although some of these findings are in conflict, the research in this section highlights both short‐ and long‐term associations between sleep regulation and vocabulary. However, these associations change depending on the specific parameters of sleep regulation being examined (e.g., number of naps, overnight sleep duration, etc.), the age of the child and nap habituality.

### 1.3 Motor Skill and Vocabulary Development

Many studies found evidence for the interconnectedness of the language and the motor systems, even from long before infants are producing words. For example Iverson and Thelen (1999), building upon evidence indicating the close relatedness of motor and language functions in the brain and the coordination of hand and mouth activity from birth (e.g., the Babkin reflex), examine evidence supporting the idea that the rhythmic limb and torso movements commonly observed in infancy, such as waving, swaying and banging, coincide with the rhythmic jaw movements that, coupled with vocalization, give rise to babbling behavior. Through this co‐activation, vocal and gestural behaviors entrain one another and eventually co‐develop toward the precise coupling of words and gestures observed in adult communication (Iverson and Thelen 1999).

The relationship between motoric and language behavior is not limited to the synchronicity between them. Reaching new motoric milestones can have cascading effects on infant communication (Campos et al. 2000). This is because mastering a new motor skill opens a new set of ways in which infants can perceive and act upon their environment, which in turn shapes the level of linguistic input and output that they receive from and direct to the environment.

The onset of independent locomotion is particularly significant in this respect, as it enables infants to reach distant objects and explore the space around them autonomously; this has important implications for cognition and communication. The onset of crawling on hands and knees marks the onset of willing, autonomous, self‐initiated locomotion; it has been referred to as the “psychological birth of the human infant” (Mahler, Pine, and Bergman 1975, cited in Campos et al. 2000). Crawling also introduces important changes in the way infants perceive the world around them and its onset is followed by dramatic changes in emotional, social, communicative and cognitive development (Campos et al. 2000). Among other things, crawling significantly increases the opportunities for social interactions, by introducing the need for distal communication and referencing. It also increases the number of referents parents and children can label and refer to (Campos et al. 2000).

The transition to independent walking similarly influences communicative advances. As infants begin to walk independently, they can start to carry objects to their caregivers with their hands; notably, they do so more frequently than their crawling same‐age peers (Karasik, Tamis‐LeMonda, and Adolph 2014). An earlier onset of the ability to sit and walk predicted larger productive vocabulary growth in 16‐ to 28‐month‐olds (Oudgenoeg‐Paz, Volman, and Leseman 2012). Concurrently, the onset of walking is associated with an increase in receptive and productive vocabulary, independently of age (Walle and Campos 2014). Infants who walk independently vocalize more and spend longer periods of time interacting with their caregiver compared to their peers who walk with the aid of a walker (Clearfield 2011). This suggests that independent locomotion specifically (and not the movement of the limbs involved in walking or the fact of moving upright through space per se) is associated with a stronger base for communication (Clearfield 2011). This is mirrored in the results from the naturalistic observations of parent‐child interactions in Walle and Campos (2014), where the level of linguistic input from the parent was significantly correlated with larger receptive and productive vocabularies in walking (but not crawling) infants. These findings suggest that level of motor skill and language input interact to shape further communicative development.

### 1.4 Sleep and Motor Skills

Finally, research has found that motor activity and sleep are also associated during development. For example, sleep has been found to support memory consolidation for motor tasks in infants. Interestingly, results in this domain seem to echo those of the effects of sleep on the consolidation of novel words. Berger and Scher (2017) demonstrated that infants who napped after motor training exhibited improved performance in a problem‐solving locomotion task compared to those who stayed awake. Moreover, DeMasi et al. (2021) found that a nap shortly following motor training was the most beneficial to learn the task most efficiently. Notably, Horger et al. (2023) showed that infants who napped within 2 h of training displayed enhanced motor performance the following day, reflecting a delayed effect of napping on memory consolidation for motor patterns similar to that observed for the consolidation of vocabulary (Belia, Keren‐Portnoy, and Vihman, in press; Hupbach et al. 2009).

Other studies suggest that motor development can influence sleep, too. In Scher and Cohen (2005), 5–8‐month‐olds who had started crawling had more night awakenings than their non‐crawler peers, regardless of age. In an actigraphy study with 8‐month‐olds, infants with higher motor scores who had already started crawling experienced more awakening episodes at night, longer sleep duration and higher levels of motor activity during sleep than non‐crawling age peers (Scher 2005). These findings provide evidence for an effect of the onset of crawling on sleep. This has been hypothesized to be due to higher energy expenditures during the day in relation to more demanding motor activity, and to more intense emotional responses in caregiver and infant alike following the emergence of independent locomotion. These factors are likely to influence levels of arousal, tiredness and stress at bedtime (Scher and Cohen 2005). Recent research suggests that this sleep disturbance may be caused by the “twitches” seen during mammalian sleep, characterized by rapid, isolated muscle movements, which might contribute to the consolidation of sensorimotor information by activating brain regions involved in motor control (Khazipov et al. 2004; Blumberg 2015; Blumberg et al. 2013; Sokoloff et al. 2021). These findings add complexity to the relationship between sleep, language and motor domains, suggesting that motor development may disrupt sleep, affecting vocabulary growth in more complex ways than previously thought.

Sleep disturbances and twitches have been studied in relation to the onset of new locomotion skills. No studies so far have investigated whether the onset of other forms of motor behavior, such as vocal production, is associated with similar disturbances in sleep.

### 1.5 This Study

The sections above summarized existing research that shows significant connections between self‐locomotion and vocabulary, sleep regulation and vocabulary, and sleep and self‐locomotion. However, to date, no studies have systematically examined these associations together, despite their individual importance in the literature.

We have two primary objectives: (a) to determine whether sleep patterns, self‐locomotion, and vocabulary development are mutually associated and (b) if so, to examine how these associations evolve over time. By examining these relationships collectively, we aim to provide a comprehensive understanding of the relationship between these three domains. We hope that this exploration will lay the groundwork for future research to delve more deeply into the mechanisms underlying vocabulary development in relation to sleep and the emergence of self‐locomotion, two major elements in an infant's life.

To achieve these objectives, we have collected longitudinal data on motor abilities, sleep habits, and vocabulary development from the same group of children at ages 7, 12, 16, and 24 months. We then analyzed whether changes in self‐locomotion and sleep habits influence vocabulary development. Our overarching goal is to provide a biologically informed understanding of vocabulary development, considering it both as a memory‐based process and the result of interactions between the body and its environment. We adopt the theoretical framework of Dynamic Systems Theory, which views development as unfolding through complex interactions among cognition, the environment, and the physical body (Thelen and Smith 1994).

### 1.6 Data Availability Statement

The data that support the findings of this study are available on the OSF database (link: https://osf.io/es83q/; DOI: https://doi.org/10.17605/OSF.IO/ES83Q).

## 2 Methods

### 2.1 Participants

A total of 89 children were initially included in the study. The sample size for this study was determined by practical constraints, specifically the time and funding available within the first author's PhD project. Some children were excluded due to families withdrawing from the study (*n* = 17) or missing two or more data collection points (*n =* 21). The final sample consisted of 51 children, 20 of whom started participating at 7 months of age, contributing four data points, while the rest joined at 1 year of age, contributing three data points. Participants were based in the UK, mostly in the Yorkshire and Humber area. They were mainly raised in mid‐to‐high income households, with a White British/Irish ethnic background and British English as the primary language. Most participants were born full‐term and all the babies weighed at least 5lbs 8oz at birth. Aside from one child with hearing problems, they had no known hearing, vision or developmental problems. Families were contacted through social media, existing participant databases and through the advertisement channels of other UK‐based developmental laboratories.

Data were collected at ages 7 months (*n =* 20, range = 6; 24–8; 7, *M* = 7; 8), 12 months (*n =* 51; range = 11; 23–13; 4, *M* = 12; 6), 16 months (*n =* 51, range = 14; 28–16; 26, *M* = 16; 1) and 24 months (*n =* 51, range = 23; 25–24; 17, *M* = 24; 8). The mean ages and ranges are based on the dates of completion of the first day of the sleep diary (see below) at each data collection point.

All procedures involving human subjects in this study were approved by the Language and Linguistic Science ethics committee at the University of York. All parents provided written informed consent for each child prior to data collection.

### 2.2 Procedure

We collected data about the children's sleep, vocabulary and motor skills at every age, using three different questionnaires.

1. Children's sleep was assessed using an adaptation of the Sleep and Naps Oxford Research Inventory (SNORI) (Horváth and Plunkett 2016). This is a paper diary where parents are asked to record their child's sleep behavior over 10 days. We shortened the diaries to 5 days, to limit participant attrition and facilitate compliance. On each day (over a 24‐h timeline) parents were asked to indicate the times when their child was asleep. They were also asked to note any night awakenings and where their child was sleeping (e.g., pushchair, car, cot). They were also asked to indicate any nursery days and any special events or conditions (e.g., severe illness, trips to countries in different time zones). Any sleep occurring between 8 a.m. and 6 p.m. was considered daytime sleep. We considered a nap to be any parent‐reported bout of sleep occurring during this time frame. Any sleep occurring between 6 p.m. and 8 a.m. was considered nocturnal sleep (see Horváth and Plunkett 2016).

Through the SNORI we collected the following data:

– Number of daytime naps (NN): any sleep bouts occurring between 8 a.m. and 6 p.m.
– Number of night awakenings (NNA): any stretches of time the child spent in an awake state between 6 p.m. and 8 a.m.
– Proportion of sleep occurring at night versus during the day (N/D). This was calculated as follows:
$$\frac{\text{Nocturnal}\;\text{sleep}\;\text{duration}-\text{Daytime}\;\text{sleep}\;\text{duration}}{\text{Nocturnal}\;\text{sleep}\;\text{duration}+\text{Daytime}\;\text{sleep}\;\text{duration}}$$

Thus N/D is a number between −1 and +1, with a value of −1 corresponding to sleep occurring exclusively between 8 a.m. and 6 p.m. and +1 corresponding to sleep occurring exclusively between 6 p.m. and 8 a.m. A value of 0 would suggest equal time spent asleep during the day and during the night. N/D values closer to +1 suggest shorter time spent asleep during the day and/or longer time spent asleep during the night.

2. Children's vocabulary was assessed via an online version of the UK Communicative Development Inventory – Words and Gestures (CDI) (Alcock et al. 2020). This inventory is a checklist of words commonly acquired between 8 and 18 months. Parents are asked to indicate which words their children understand only and which ones they also say. Scores for receptive and productive vocabulary are derived by counting how many words the parent reported their child to understand and to both understand and say. Specifically, receptive vocabulary size is calculated as the sum of the “understands” and “understands and says” scores, whereas the productive vocabulary size corresponds to the “understands and says” score alone.
3. Motor skills were assessed via the Vineland Adaptive Behavior Scales (Motor Skill Domain) (Sparrow and Cicchetti 1989). We used only the form intended to be filled in by parents/caregivers; we adapted this to an online format. The form comprises a list of actions and motor abilities of different levels of complexity (e.g., from sitting or beginning to stand or walk, to climbing up and down the stairs or completing puzzles) and sub‐actions (e.g., sitting unsupported for at least 1 min, taking at least two steps, etc.). Caregivers were asked to indicate the frequency with which their child carried out each specific action, selecting from the following options: “Almost always,” “Often,” “Sometimes,” “Rarely” and “Never.” They were also asked to mark as “Almost always” any skills that the child used to exhibit but that they had grown out of. So that we could treat the variable as numerical in our analyses, we assigned the following numerical values to each response category: Almost always = 5; Often = 4; Sometimes = 3; Rarely = 2; Never = 1. For the purposes of this study, we examined the two motor variables associated with independent locomotion: crawling and walking. We adopted conservative criteria to code for these two behaviors. Crawling was intended to mean locomoting across the floor on hands and knees, without the abdomen touching the ground, for at least 5 feet. Similarly, we considered only stable walking behavior, unsupported and employed by the child as their most common form of locomotion.

## 3 Results

### 3.1 Descriptive Analysis

#### 3.1.1 Sleep Variables

**FIGURE 1:**
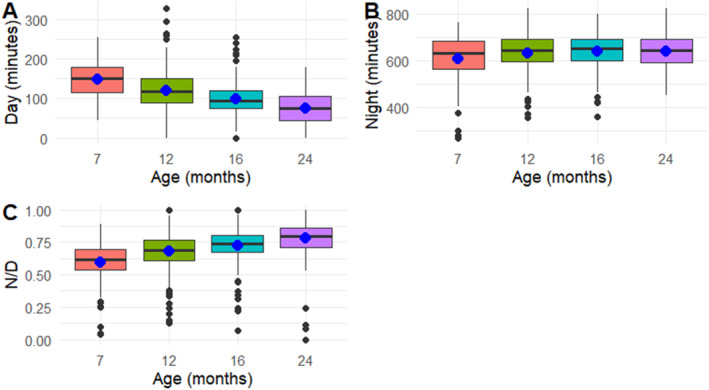
Average and median sleep duration (in minutes) during the day (A) and during the night (B) across age, with N/D values across age (C). The average score is indicated with a blue dot.

Figure 1 shows an approximately constant night‐time sleep duration across age, but a decrease in daytime sleep. Figure 1 also shows that overnight sleep proportion (N/D) increases with age, progressively approaching 1 across the four data collection points.

Two Kruskal–Wallis tests were carried out to assess whether night‐time and daytime sleep duration were significantly different by age. Age had a significant, relatively strong effect on daytime sleep duration (*χ* ^2^(3, *N* = 852) = 177.13, *p* < .001, *ε* ^2^ = .20). In contrast, the effect of age on night‐time sleep duration was not significant (*χ* ^2^(3, *N* = 852) = 6.51, *p* = 0.09, *ε* ^2^ = .004).

**FIGURE 2:**
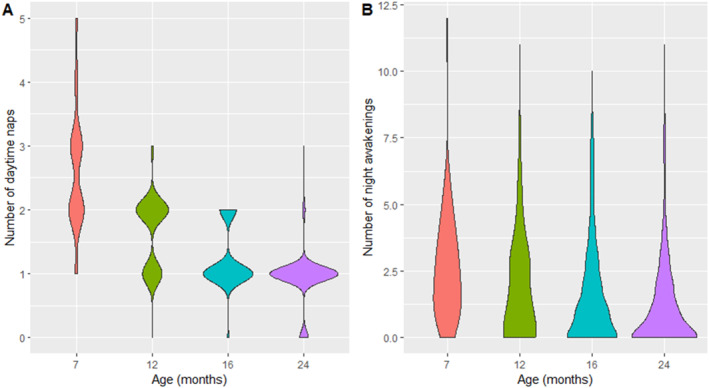
Number of naps (A) and number of night awakenings (B) across age.

Figure 2 displays the number of naps and night awakenings across age, showing that both parameters decrease with age.

#### 3.1.2 Vocabulary Size

**FIGURE 3:**
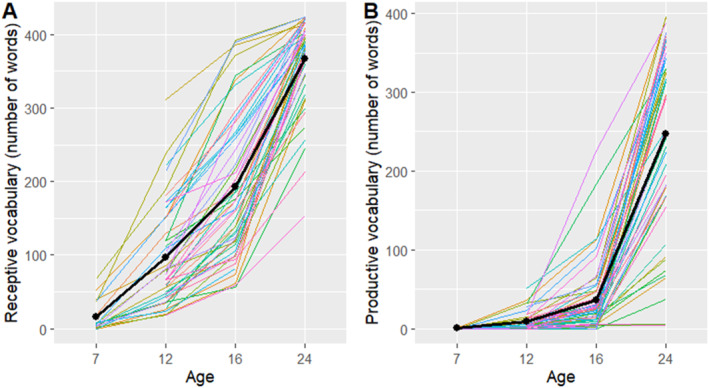
Receptive vocabulary (A) and productive vocabulary (B) across age. Colored lines are individual participants. The black line connects the means of the individual trajectories.

Figure 3 shows the growth of receptive and productive vocabulary across age.

#### 3.1.3 Motor Skills

**FIGURE 4:**
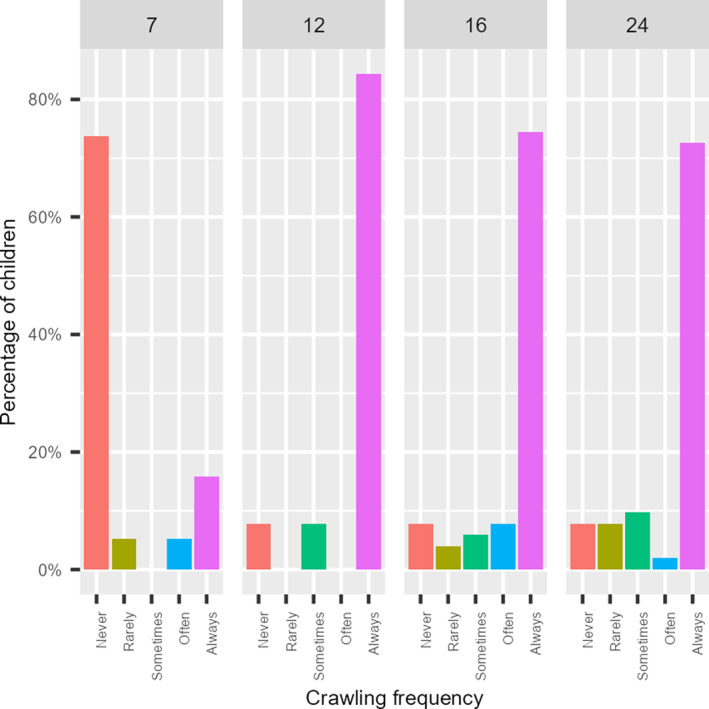
How often children were reported to crawl at each age.

**FIGURE 5:**
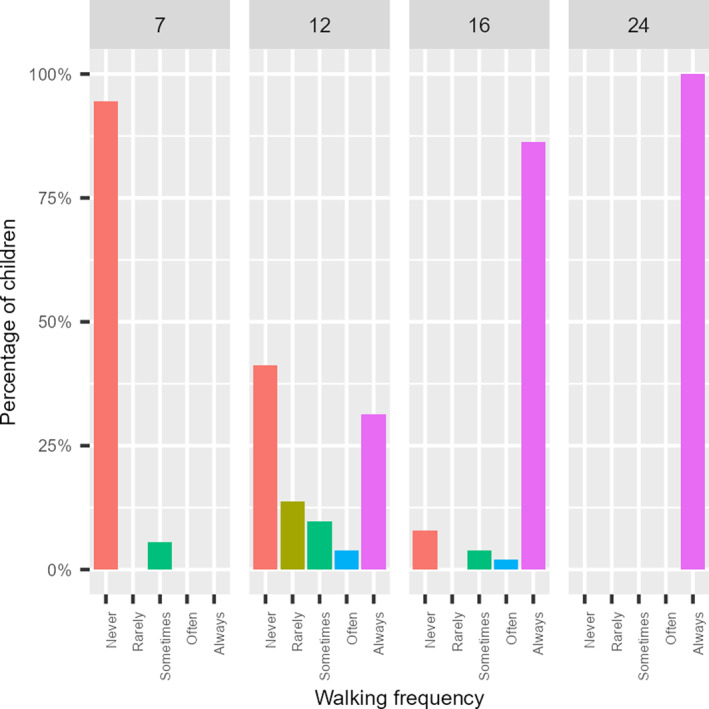
How often children were reported to walk at each age.

Table 1 describes how frequently children were reported to be walking and crawling as a way to locomote at each age, while Figures 4 and 5 represent the percentage of children categorized by the frequency of crawling or walking at each age, ranging from those who never crawled to those who crawled rarely, sometimes, often, or almost always.

**TABLE 1:** Frequency of crawling and walking behavior at each age (almost always = 5; often = 4; sometimes = 3; rarely = 2; never = 1).

Age	Behavior	Mean (SD)	Median	Range	SE
7	Crawling	1.842 (1.538)	1	1–5	0.158
	Walking	1.111 (0.461)	1	1–3	0.049
12	Crawling	4.529 (1.163)	5	1–5	0.073
	Walking	2.706 (1.733)	2	1–5	0.109
16	Crawling	4.373 (1.239)	5	1–5	0.078
	Walking	4.588 (1.126)	5	1–5	0.07
24	Crawling	4.235 (1.34)	5	1–5	0.084
	Walking	5 (0)	5	n/a	0

### 3.2 Main Analysis

We wanted to understand whether the development of sleeping patterns and the motor skills associated with independent locomotion have a combined influence on vocabulary development, whether receptive, productive or both. Accordingly, we ran two mixed effects models with sleep and motor development as independent variables and vocabulary as the dependent variable.

#### 3.2.1 Correlations Between Sleep Parameters

To improve the interpretability of our mixed effects model, we checked the correlation between the sleep variables before inputting them into the model. The results of these correlations are shown in Table 2.

**TABLE 2:** Correlations between number of naps (NN), number of night awakenings (NNA) and the night over daytime sleep ratio (N/D) per age.

Age	Correlation	df	*R*
7	NN ∼ NNA	82	0.12
	NN ∼ N/D	82	−0.5***
	NNA ∼ N/D	82	−0.44***
12	NN ∼ NNA	207	−0.08
	NN ∼ N/D	207	−0.27***
	NNA ∼ N/D	207	0.12
16	NN ∼ NNA	242	0.044
	NN ∼ N/D	242	−0.25***
	NNA ∼ N/D	242	0.027
24	NN ∼ NNA	238	−0.027
	NN ∼ N/D	238	−0.29***
	NNA ∼ N/D	238	−0.11

Significance codes: ***p ≤ 0.001, **0.001 ≤ *p* ≤ 0.01, *0.01 ≤ *p* ≤ 0.05.

Number of naps (NN) correlated with nocturnal sleep proportion (N/D) at all ages, and number of night awakenings (NNA) correlated with N/D at 7 months. Therefore, only N/D was included as a sleep variable in the model. This variable provides information about both daytime and night‐time sleep, contrary to NN (daytime sleep only) and NNA (night‐time sleep only).

#### 3.2.2 Statistical Models

The descriptive statistics identified subsets of data of varying informativeness in terms of variance. Figure 4 suggests that almost all 12‐month‐olds in our sample were reported to “almost always” crawl when moving around, or that they had grown out of crawling at that age. Similarly, Figure 5 shows a sharp increase in walking frequency between 12 and 16 months, culminating in a plateau at 24 months. Thus data about crawling frequency at 12, 16, and 24 months are unlikely to be informative as they contribute little variance; virtually all the children were reported to be crawling consistently at those ages. This applies to data about walking frequency at 7 months, when virtually no infant was reported to be walking. Similarly, the number of words produced at 7 months is virtually zero (Figure 3). Therefore, productive vocabulary at 7 months is likely to be relatively uninformative. On the other hand, overnight sleep proportion never exhibited floor or ceiling effects, and was therefore treated as informative at all ages in the study.

Given the trends described above, we divided the dataset into two subsets: 7–16 months of age, for which we investigated the effects of overnight sleep proportion and crawling frequency on receptive vocabulary, and 12–24 months of age, for which we investigated the effects of overnight sleep proportion and walking frequency on productive vocabulary. Based on this, we built and ran two separate mixed effects models. Their results are compared in the Discussion section. In addition, both models were run on the square root‐transformed vocabulary data. This kind of transformation was applied to both receptive and productive vocabulary to improve the normality of their distribution, given the different degrees of variability in the vocabulary data observed across age. Therefore, the coefficients, statistical significance and figures reported in the subsequent analyses pertain to the transformed data.

Statistical analysis was run in R (R Core Team 2015). We conducted both linear mixed effects regression models using the lme4 package (Bates et al. 2015). The first model (run with data from children aged 7–16 months) investigated receptive vocabulary size as the dependent variable, while overnight sleep proportion (N/D) and crawling frequency constituted the predictor variables. In the second model (run with children aged 12–24 months), productive vocabulary size was the dependent variable and overnight sleep proportion and walking frequency were the predictor variables. Analyses of each model are detailed in what follows.

##### 3.2.2.1 Model 1—The Effect of Crawling Frequency and Overnight Sleep Proportion in Receptive Vocabulary Between 7 and 16 months

The first model was run with a sample size of 20 7‐month‐olds, 51 12‐month‐olds and 51 16‐month‐olds. It tested the effects of crawling frequency (*crawling*) and N/D (*nd*) on receptive vocabulary (*rec.voc*). To account for the fact that the effects of N/D and crawling frequency could vary by age, we included interaction terms between age and the predictors of interest N/D (*nd × age*) and crawling frequency (*crawling × age*). As the study involved repeated measures per participant, we inputted the random effect for the variable *participant* in the model. The resulting full model was the following:

rec.voc∼nd×age+crawling×age+(1|participant)

To assess the adequacy of our model in explaining the variation in receptive vocabulary, we conducted likelihood ratio tests to assess the goodness of fit of our model compared to nested models. Including age (*χ* ^2^(2) = 108.72, *p* < 0.001) and crawling frequency significantly improved the model fit (*χ* ^2^(1) = 42.56, *p* < 0.001). N/D alone did not improve the model fit (*χ* ^2^(1) = 2.58, *p* = 0.108). The interactions of N/D and crawling with age significantly improved the model fit (*crawling × age*: *χ* ^2^(2) = 7.99, *p* = 0.018; *N/D × age*: *χ* ^2^(2) = 21.52, *p* < 0.001). This analysis suggests that the interaction terms capture additional variation beyond the main effects alone and, therefore, both are inputted in the model. The full model provided a strong overall fit to the data, explaining a substantial proportion of the variability in receptive vocabulary size. Fixed and random effects combined accounted for approximately 96% of the variability in receptive vocabulary (conditional *R* ^2^ = 0.959). The fixed effects alone predicted a smaller proportion of the variance in receptive vocabulary, as indicated by the marginal *R* ^2^ value of 0.491. Nevertheless, this value suggests that fixed effects still contributed to approximately 49% of the variability in receptive vocabulary.

The likelihood ratio test revealed that the full model was the best at explaining the vocabulary data, so the full model was also the final model that we conducted (i.e., rec.voc ∼ nd × age + crawling × age + (1 | participant)). Test statistics (see Table 3) revealed significant effects on receptive vocabulary size of several individual predictors in the full model. As expected, children aged 12 and 16 months had a significantly larger vocabulary compared to the reference age group (7 months). Negative main effects on receptive vocabulary size were found of crawling frequency and N/D. However, we found increases in receptive vocabulary size per one‐unit increase in crawling frequency both at 12 and at 16 months. A similar pattern was identified for N/D at 12 and 16 months. See Table 3 for the full test statistics.

**TABLE 3:** Linear mixed model results with crawling frequency, N/D and their interactions with age as the fixed effects and receptive vocabulary size as the dependent variable.

Term	Estimate	SE	*t* (df)	*p*	95% CI lower	95% CI upper
N/D	−3.051	0.757	−4.033 (546.538)	< 0.001	−4.535	−1.57
Crawling	−0.489	0.0820	−5.958 (546.682)	< 0.001	−0.649	−0.3279
Age 12	3.343	0.604	5.534 (544.190)	< 0.001	2.166	4.521
Age 16	6.661	0.621	10.734 (544.314)	< 0.001	5.451	7.87
Crawling × age 12	0.211	0.100	2.106 (546.445)	0.035	0.016	0.406
Crawling × age 16	0.285	0.102	2.786 (547.049)	0.006	0.086	0.484
N/D × age 12	3.273	0.854	3.833 (544.704)	< 0.001	1.607	4.936
N/D × age 16	3.896	0.862	4.521 (544.394)	< 0.001	2.216	5.576

**FIGURE 6:**
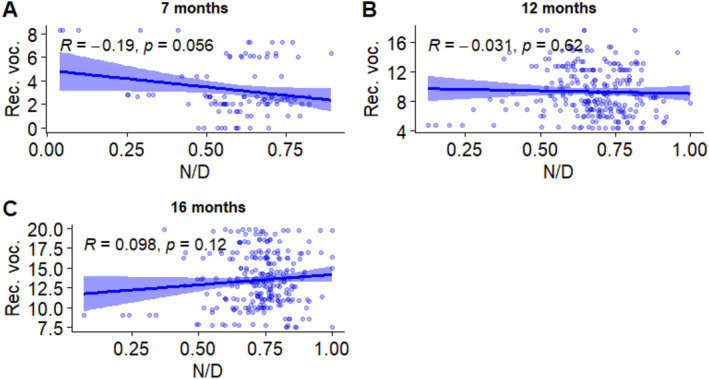
Correlations between overnight sleep proportion and receptive vocabulary at 7 months (A), 12 months (B), and 16 months (C). The regression line represents the estimated relationship between overnight sleep proportion and vocabulary, with 95% confidence intervals depicted around the regression line. Pearson correlation coefficient (*R*) shows the strength and direction of the relationship.

**FIGURE 7:**
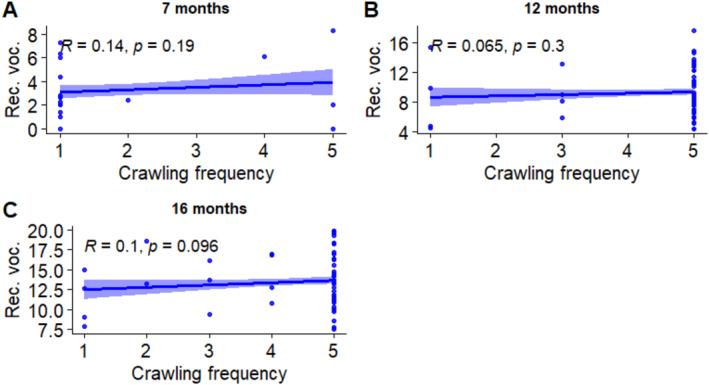
Correlations between crawling frequency and receptive vocabulary at 7 months (A), 12 months (B), and 16 months (C). The regression line represents the estimated relationship between crawling frequency and vocabulary, with 95% confidence intervals depicted around the regression line. Pearson correlation coefficient (*R*) shows the strength and direction of the relationship.

Overnight sleep proportion and crawling each have individual effects on receptive vocabulary in the model. However, interactions of age with both overnight sleep proportion and crawling were significant, suggesting that these two variables were associated in different ways with vocabulary at specific ages. Correlations between the two predictor variables and receptive vocabulary at each age were measured to further investigate this finding. The results are shown in Figures 6 and 7.

We found one marginally significant correlation between overnight sleep proportion and receptive vocabulary size at 7 months, which was relatively weak and negative. No significant correlations were found between receptive vocabulary and crawling.

##### 3.2.2.2 Model 2—The Effect of Walking Frequency and Overnight Sleep Proportion on Productive Vocabulary Between 12 and 24 months

The second model was run with a sample size of 51 12‐month‐olds, 51 16‐month‐olds and 51 24‐month‐olds. It investigated whether walking frequency (*walking*), and N/D (*nd*) influenced productive vocabulary (*prod.voc*) in infants aged 12–24 months. As in the first model, interaction terms between age and the predictors (*nd × age* and *walking × age*) were included. Similarly, *participant* was treated as a random effect. The initial full model was the following:

prod.voc∼nd×age+walking×age+(1|participant)

Again, to assess the adequacy of our model in explaining variation in productive vocabulary, we conducted likelihood ratio tests. The full model significantly outperformed another model lacking the *walking × age* interaction term (*χ* ^2^(1) = 6.29, *p* = 0.012). The *nd × age* interaction, as well as the single predictors of age and N/D did not significantly improve the model fit (*nd × age*: *χ* ^2^(2) = 0.29, *p* = 0.866; age: *χ* ^2^(2) = 1.53, *p* = 0.216; N/D: *χ* ^2^(2) = 1.68, *p* = 0.195). The contribution of walking as an individual predictor was not estimable (*χ* ^2^(0) = 0, *p* = NA). Given that the interaction between N/D and age did not contribute to explaining the variability in productive vocabulary, we only included the interaction between walking and age in the final model we conducted:

prod.voc∼walking×age+(1|participant)

Overall, this model strongly fitted our data, explaining approximately 90% of the variability in productive vocabulary (conditional *R* ^2^ = 0.893). While fixed effects alone predicted a slightly smaller portion of the variance (marginal *R* ^2^ = 0.73), they still accounted for 73% of the variability in productive vocabulary.

The test statistics of this model are available in Table 4. As expected, 24‐month‐olds had a significantly larger vocabulary compared to the reference age group (12 months), but productive vocabulary size was not statistically different between 12 and 16 months. We found a positive effect on productive vocabulary of the interaction between age and walking frequency at 16 months. This suggested a significant increase in estimated productive vocabulary as walking frequency increased at 16 months, compared to the 12 months reference age group.

**TABLE 4:** Linear mixed model results with walking frequency, N/D and their interactions with age as the fixed effects and productive vocabulary size as the dependent variable.

Term	Estimate	SE	*t* (df)	*p*	95% CI lower	95% CI upper
Walking	−0.061	0.093	−0.65 (729.524)	0.516	−0.264	0.102
Age 16	0.857	0.706	1.214 (724.693)	0.225	−0.822	3.626
Age 24	12.932	0.284	45.542 (717.613)	< 0.001	11.254	16.354
Walking × age 16	0.442	0.161	2.739 (721.476)	0.006	0.092	0.743

**FIGURE 8:**
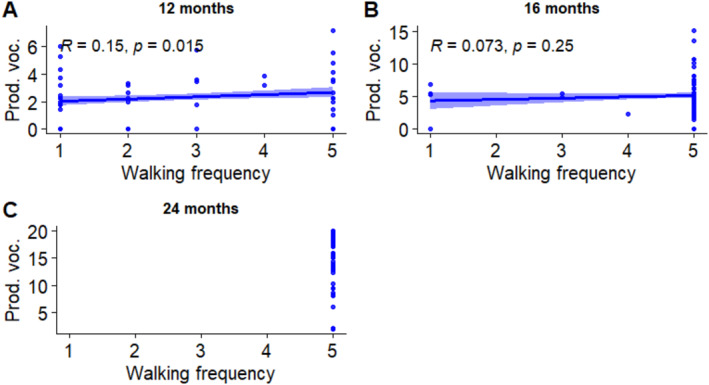
Associations between walking frequency and productive vocabulary at 12 months (A), 16 months (B), and 24 months (C). The regression line represents the estimated relationship between walking frequency and vocabulary, with 95% confidence intervals depicted around the regression line. Pearson correlation coefficient (*R*) shows the strength and direction of the relationship.

The interaction between age and walking was significant, suggesting that these two variables are differently associated with productive vocabulary at specific ages. Correlations between walking and productive vocabulary at each age were measured and their results are represented in Figure 8.

We found a weak but positive and significant correlation between productive vocabulary and walking frequency at 12 months.

### 3.3 Additional Analyses

Based on the literature regarding the impact of self‐locomotion skills on sleep patterns, we analyzed the relationship between sleep variables and locomotion skill within the 7‐ and 12‐month age groups. The descriptive analysis revealed a considerable increase in crawling behavior between 7 and 12 months (Figure 4), as well as a marked increase in walking frequency between 12 and 16 months (Figure 5). While our measure of self‐locomotion may not have precisely captured the onset of these skills, these findings suggest that crawling and walking likely emerged at 7 and 12 months, respectively, in our sample. Although we are limited in the extent to which we can definitely interpret any associations between sleep patterns and the onset of these locomotion skills, exploring these relationships could provide valuable insights for interpreting the results of our main analyses.

**FIGURE 9:**
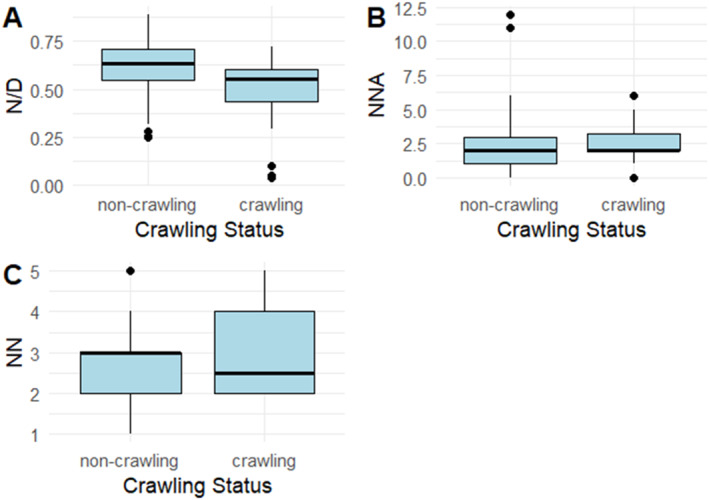
Overnight sleep proportion (A), number of night awakenings (B) and number of naps (C) in 7‐month‐olds who consistently crawl (i.e., crawling frequency = “Often” or “Almost always”) versus those who do not consistently crawl (i.e., crawling frequency = “sometimes,” “Rarely” or “Never”).

First, we examined the number of naps (NN), the number of night awakenings (NNA) and the overnight sleep proportion (N/D) of 7‐month‐olds who consistently crawled (i.e., crawling frequency = “Often” or “Almost always”) and those of infants who did not consistently crawl (i.e., crawling frequency = “never,” “Rarely” or “Sometimes”). The two groups of infants are compared in Figure 9.

A Wilcoxon test revealed that overnight sleep proportion was significantly smaller in crawling 7‐month‐olds (*n* = 4) compared to non‐crawling ones (*n* = 15) (*W* = 1063.5, *p* = 0.003), suggesting that the former slept proportionately more during the day than the latter. The two groups did not differ in terms of number of night awakenings (*W* = 675.5, *p* = 0.547) or number of naps (*W* = 658, *p* = 0.367).

**FIGURE 10:**
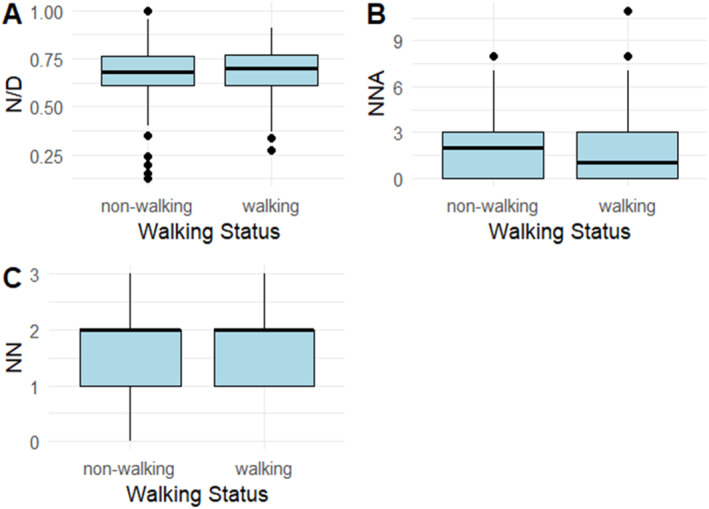
Overnight sleep proportion (A), number of night awakenings (B) and number of naps (C) in 12‐month‐olds who consistently walk (i.e., walking frequency = “Often” or “Almost always”) versus those who do not consistently walk (i.e., walking frequency = “sometimes,” “Rarely” or “Never”).

We then compared the sleep variables of 12‐month‐olds reported to be consistently walking (i.e., walking frequency = “Often” or “Almost always”) and those not yet consistently walking at that age (i.e., walking frequency = “never,” “Rarely” or “Sometimes”). The results are shown in Figure 10.

A Wilcoxon test revealed no significant differences in any of the sleep parameters between walking (*n* = 23) and non‐walking (*n* = 28) 12‐month‐olds (N/D: *W* = 7818, *p* = 0.782; NNA: 8467.5, *p* = 0.392; NN: *W* = 8000, *p* = 0.969).

In the Background section, we discussed how vocal production entails a motor component, involving the fine coordination of muscles in the articulators, and presented studies identifying entrainment between the vocal and motor domain during development (e.g., Iverson and Thelen 1999). We highlighted the fact that vocal production has never been investigated in relation to disruptions in the sleep variables associated with motor learning. The first step to test this would be to assess the extent to which development in the motor system, beyond locomotion, is associated with the development of vocal production.

**FIGURE 11:**
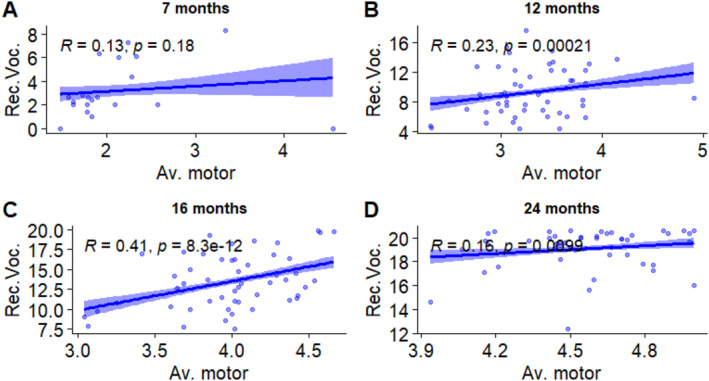
Associations between receptive vocabulary size (square‐root transformed) and average motor score at 7 months (A), 12 months (B), 16 months (C), and 24 months (D). The regression line represents the estimated relationship between general motor score and vocabulary, with 95% confidence intervals depicted around the regression line. Pearson correlation coefficient (*R*) shows the strength and direction of the relationship.

**FIGURE 12:**
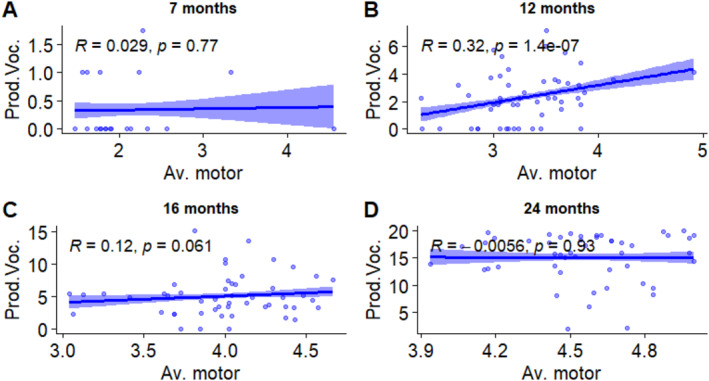
Associations between productive vocabulary size (square‐root transformed) and average motor score at 7 months (A), 12 months (B), 16 months (C), and 24 months (D). The regression line represents the estimated relationship between general motor score and vocabulary, with 95% confidence intervals depicted around the regression line. Pearson correlation coefficient (*R*) shows the strength and direction of the relationship.

To do this, we first calculated a general motor score based on the responses to the Vineland Adaptive Behavior Scales (Motor Skill Domain) (*av.mot*) and used it as a proxy for general level of motor development. Then, we tested the associations between this variable and receptive vocabulary, which does not entail motor activity, and productive vocabulary, which entails a motor component in the articulation of words. Figures 11 and 12 show the outcomes of this analysis.

At 7 months, the correlation between *av.mot* and receptive vocabulary was not significant. At 12 months it was significant and positive, although relatively weak. At 16 months, the association was still significant and positive but of an increased magnitude. At 24 months, the association was still significant but weak.

At 7 months, the correlation between *av.mot* and productive vocabulary was not significant. It was significant, positive and moderately strong, at 12 months. At 16 months, the association was weak and only marginally significant. At 24 months, the association was not significant.

## 4 Discussion

### 4.1 Summary of Results

While previous studies have noted links between the development of sleep regulation, self‐locomotion and vocabulary growth, their interconnectedness has never been systematically investigated. This study addressed this gap by exploring whether and how these three domains are related to each other, by viewing vocabulary growth as a holistic and embodied process intimately tied to the growth of memory networks and, at the same time, in close connection with the on‐going interaction between the developing body and the environment.

The results from Model 1 suggest a complex relationship between crawling frequency, overnight sleep proportion and receptive vocabulary between 7 and 16 months. Overall, both crawling and overnight sleep had negative main effects, meaning that infants who crawled more frequently (or had stopped crawling) and those who slept proportionally more overnight tended to have smaller receptive vocabularies at each of the measured ages (7, 12, and 16 months). However, the positive effect of the interaction between these variables and age reveals a more detailed story: Infants who either crawled more or had outgrown crawling and slept more at night showed significant increases in receptive vocabulary at 12 and 16 months, compared to when they were 7 months old. This indicates that while sleep and crawling were not positively associated with receptive vocabulary at the younger ages we examined, their relationship with vocabulary growth appeared to become more positive as the infants got older. In Model 2, walking frequency at 16 months showed a strong positive effect on productive vocabulary growth compared to when the infants were 12 months old, but had no main effects. This indicates that walking frequency was positively associated with productive vocabulary growth over time only when considered in relation to the infants' age.

We further explored these findings through correlation analyses (Figures 6, 7, 8). Our findings, although modest in magnitude, show that 12‐month‐olds who walked more frequently had larger productive vocabularies. In addition, 7‐month‐olds sleeping proportionately more at night tended to have smaller receptive vocabularies, although this was only a marginally significant correlation.

Finally, two additional analyses explored the associations between sleep and motor development. In the first analysis, we examined the sleep patterns of infants at ages corresponding to the emergence of crawling (around 7 months) and walking (around 12 months) in our data. We found that 7‐month‐olds who were reported to crawl more slept proportionally less at night compared to their non‐crawling peers. In the second analysis, we explored the basis for treating the motor component involved in word production as a motor skill whose onset can potentially affect sleep patterns, much as the onset of self‐locomotion has been thought to do. A significant and moderately strong association was found between productive vocabulary and overall motor score at 12 months, while receptive vocabulary positively correlated with overall motor development at 12, 16, and 24 months.

### 4.2 The Relationship Between Sleep, Motor and Vocabulary Domains Are Not Driven Only by Age

As expected, in this study, children's vocabulary increased significantly overall with age. One or both of the main predictors—sleep and motor variables— interacted significantly with age in each model. Thus, (a) the relationship between self‐locomotion, sleep regulation and vocabulary exhibited nonlinear changes over time and (b) locomotion and overnight sleep proportion had effects on vocabulary at certain ages but not others. Our results support the hypothesis that maturation alone does not explain vocabulary growth; rather, it could be influenced by sleep and motor development at different points in time.

### 4.3 Locomotion Effects on Vocabulary

Although it had a negative main effect, crawling frequency predicted a significant increase in receptive vocabulary size at 12 and 16 months compared to the 7‐month baseline (Model 1). Walking at 16 months predicted an even bigger increase in productive vocabulary size, compared to the 12‐month‐old baseline (Model 2). Furthermore, walking frequency positively correlated with productive vocabulary at 12 months, but no other associations were found between self‐locomotion and vocabulary at any other ages. These results could be explained in different ways.

First, these associations may have emerged at these ages due to the timing of self‐locomotion skill development. Crawling and independent walking begin to appear around 7 and 12 months, respectively, in the kind of population we investigated (Størvold, Aarethun, and Bratberg 2013; Bayley 1969; Frankenburg et al. 1992, as reported in Adolph and Robinson 2015). Our descriptive analysis confirms this, with 20% of 7‐month‐olds reported to be crawling and 45% of 12‐month‐olds reported to be walking, indicating that these motor skills were emerging at those ages. According to Dynamic Systems Theory (Thelen and Smith 1994), it is during periods of instability that changes in one system are more likely to prime changes in another, resulting in stronger associations between development in the two domains during periods of instability. At 12 months, some infants might still be relying on crawling to move around. At 16 months, walking behavior is still unstable, but crawling has been almost completely phased out. This could explain the stronger positive effects of walking compared to crawling on vocabulary growth at 16 months (Model 2) and the significant positive effect of crawling on vocabulary growth at 12 months (Model 1), as well as the positive correlation between walking frequency and vocabulary size at 12 months. This could also explain the absence of any effects of either self‐locomotion skill at 24 months, when walking is typically well‐established. The dynamic system interpretation of these results is further reinforced by the patterns of productive vocabulary development, which follow a similar stabilization route to the development of walking, interestingly: like walking, word production represents a relatively new and “unstable” behavior in the lives of 12‐ to 16‐month‐olds, which are also the ages where associations between these two behaviors emerge more clearly in our analyses.

Second, different forms of locomotion might relate to language ability differently. Crawling involves a posture with a more restricted visual field, primarily directed toward the floor. As hands and arms are involved in locomotion, it also limits children's opportunities to reach and engage with objects in their surroundings. This could restrict the range of referents available for joint attention episodes and labeling. In contrast, independent walking entails a larger visual field and frees up the hands, allowing children to engage more freely with objects and bring them to caregivers (Karasik, Tamis‐Lemonda, and Adolph 2014). Walking may facilitate more linguistically salient social interactions, leading to larger effects on vocabulary. This would be consistent with the stronger effects of walking on vocabulary observed in Model 2 and with the findings of the correlation analyses which, in our study, were significant for walking only at 12 months.

Interestingly, our additional analysis revealed a pattern suggesting that different forms of locomotion might also show varying associations with sleep patterns. Seven‐month‐old crawlers slept proportionately less at night than non‐crawlers, whereas there was no difference in overnight sleep proportion between 12‐month‐old walkers and non‐walkers. Considering the literature on sleep disruptions linked to the onset of locomotion, this differing relationship between crawling, walking and sleep patterns could be attributed to the significant locomotor change represented by crawling; walking may not be as arousing or tiring as crawling (Scher and Cohen 2005). Additionally, walking may not require the consolidation of different neural pathways, resulting in twitching during sleep, as crawling does (Blumberg 2015). Consequently, the transition to walking may not show as strong an association with changes in sleep patterns as the transition to crawling. We are limited in the extent to which we can interpret any associations between sleep patterns and the onset of these locomotion skills in our study, as our findings are largely correlational. In addition, our 7‐month‐old sample was relatively small and our motor skill measures were not designed to specifically capture the onset of the locomotion skill. We think a finer‐grained, larger‐scale investigation specifically monitoring the emergence of the effects of walking and crawling and comparing their relative effects on sleep patterns could clarify this matter (see Berger and Moore 2021).

There could be alternative explanations to our findings. For example, it could be argued that the positive associations between self‐locomotion and vocabulary size observed at 7 and 12 months might be more directly due to the infants' overall developmental status. This is because crawlers and walkers in our 7‐ and 12‐month age groups, respectively, are particularly advanced in self‐locomotion, as we measured crawling and walking in a conservative way, considering only crawling on hands and knees and unsupported walking. So, in this interpretation, the correlations are due to infants who confidently crawl or walk at 7 and 12 months also being particularly advanced motorically and, potentially, in other domains as well; their vocabulary might be larger than their non‐crawling or non‐walking peers because they are more advanced in general, rather than as a result of enhanced social interactions.

The method of assessing vocabulary might also have contributed to the observed differences. Parents may be more easily able to report productive vocabulary, or the number of words the child is using, compared to receptive vocabulary, or number of words the child understands. This could lead to a less precise measure that could mask the effects of crawling on receptive vocabulary and lead to apparently contradictory results, such as the negative main effect of overnight sleep proportion on receptive vocabulary (Model 1). Additionally, infants generally have a smaller productive vocabulary than receptive vocabulary, allowing more room for growth. This might make associations with productive vocabulary easier to detect, as seen in the stronger effects of walking at 16 months compared to crawling, in our models (Walle and Campos 2014). Although we observed significant main effects for crawling but not for walking, these could be attributed to the lack of variation in walking frequency at 24 months in Model 2 (see Figure 5).

### 4.4 Sleep and Vocabulary

The significant growth in receptive vocabulary observed in 12‐ and 16‐month‐olds who slept proportionately more at night contradicts the negative main effects of overnight sleep proportion in Model 1 and the absence of any effects of this variable in Model 2. We believe these findings may be only superficially contradictory. They may suggest that associations between nocturnal sleep and vocabulary vary with age, and that the first year marks a shift in the developmental trajectory of these associations.

Before year one, the consolidation of vocabulary requires more sleep throughout the day as infants' memory systems are less mature (Esterline and Gómez 2021; Gómez and Edgin 2015, 2016). By 12 months, the brain structures involved in circadian rhythm regulation and adult‐like sleep cycles have become more mature (Ednick et al. 2009), allowing infants to gradually accumulate sleep pressure more slowly and remain awake for longer periods (Esterline and Gómez 2021; Kurth et al. 2016). As infants progress into later infancy, those who sleep more at night may start to benefit more from complete and interleaved sleep cycles compared to infants who sleep less during the night and more during the day (Werchan, Ji‐Soo, and Gómez 2021; Pisch, Wiesemann, and Karmiloff‐Smith 2019). In other words, consolidated nocturnal sleep seems to become progressively more influential for memory and vocabulary learning, initially in combination with daytime sleep, and then as a primary opportunity for consolidation as children transition away from daytime napping, particularly in cultural and demographic contexts where this transition regularly occurs.

In our sample, children younger than 1 year of age who slept proportionately more at night tended to sleep less during the day. However, naps are known to support memory consolidation, including memory for new words. Considering the research reviewed in the paragraph above, such a sleep pattern may have been associated with challenges for the younger infants in our sample. For the older infants, who were further along in transitioning to consolidated nocturnal sleep, less daytime sleep may not have been as strongly linked to slower vocabulary development. This is reflected in the negative relationship observed between overnight sleep proportion and receptive vocabulary growth in Model 1, as well as the negative correlation between these variables at 7 months. As the proportion of overnight sleep of the children in our sample increased, the consolidation of their receptive vocabulary may have started to benefit from more consolidated nocturnal sleep—with positive associations starting to emerge at ages 12 and 16 months.

This idea is consistent with studies showing that the memory benefits of a post‐encoding nap are stronger in children who still habitually take naps, as their memory might also be most negatively affected by long wake intervals following learning (Riggins and Spencer 2020; Kurdziel, Duclos, and Spencer 2013). The children studied by Kurdziel, Duclos, and Spencer (2013) were aged 3–5 years, much older than the children in our study. Therefore, we are not suggesting that the older children in our sample no longer needed naps. What we are suggesting is that we might have captured the moment when infant vocabulary starts to benefit more from consolidated nocturnal sleep. This might be related to two phenomena occurring around the first birthday: (a) electrophysiological activity associated with memory and learning starting to become more organized and (b) maturation of brain areas closely linked to sleep‐wake regulation (see Ednick et al. 2009).

### 4.5 Sleep, Motor Behavior and Vocabulary: Future Directions

As mentioned in the previous section, the negative effects of overnight sleep proportion as an individual predictor on receptive vocabulary at earlier ages could be explained by the hypothesis that frequent daytime napping plays a role in the early stages of lexical development, by initiating the consolidation of newly acquired linguistic information in still‐developing memory systems. However, another line of evidence points to a connection between the onset of new locomotion skills and sleep disturbances, particularly night awakenings (Scher 2005; Scher and Cohen 2005). These, in turn, might prompt compensatory behaviors, such as waking up later in the morning or increased daytime napping (Berger and Moore 2021). This suggests an alternative explanation for the negative effects of overnight sleep proportion on early receptive vocabulary found in Model 1. In this scenario, smaller proportions of nocturnal sleep may actually be the result of sleep disruptions following the onset of a new motor skill; in this case, it would be the onset of the skill that temporarily affects overnight sleep proportion and, consequently, vocabulary consolidation.

The data from our study, however, cannot fully address this puzzle. As mentioned, our findings highlight associations across domains but do not directly indicate causal links. In addition, we collected data at ages where crawling and walking behavior were emerging, but we did not track the emergence of these skill in a fine‐grained way. However, we noted that, although based on a very small sample size, proficient crawlers at 7 months tended to have smaller proportions of overnight sleep, which in turn were negatively associated with receptive vocabulary size at the same age. Notably, these early crawlers did not show significantly more daytime napping or night awakenings than their non‐crawling peers, indicating that their sleep might not have been substantially disrupted. Nevertheless, it is essential to acknowledge that our study relied solely on parent‐reported sleep measures, which might have overlooked night awakenings where infants self‐soothed back to sleep. Therefore, we cannot entirely discount the possibility that it is the onset of crawling itself, rather than overnight sleep proportion, that temporarily affects vocabulary consolidation.

It would be of interest for future research to investigate this issue. For instance, a fine‐grained study could involve collecting data before, during and after the onset of crawling, along with more frequent assessments of sleep patterns. Sleep activity could be recorded using methods like actigraphy, video‐recordings, or polysomnography. These methods make it possible to measure sleep disruptions objectively and visualize the occurrences of twitches, which current literature suggests may be linked to the consolidation of new motor abilities but can also disrupt sleep (Sokoloff et al. 2021; Berger and Moore 2021). Such a study could clarify the relationship between sleep disruptions, crawling proficiency, and whether they exert combined effects on vocabulary.

Our data collection points were relatively widely spaced, although well placed to capture moments of important change in the domains investigated. Future large‐scale studies would do well to employ a finer‐grained data collection schedule to better understand how the relationship between sleep, self‐locomotion and vocabulary unfolds over time. Additionally, future studies could corroborate parental reports with actigraphy measures. Although our sleep data align with existing literature on sleep patterns in infancy—showing a decrease in both nap frequency and night awakenings with age (Iglowstein et al. 2003), and an overnight sleep proportion that depends more on decreased daytime sleep than increased night‐time sleep (e.g., Paavonen et al. 2020)—parent‐reported night awakenings might not reflect the actual number of times the child awoke during the night, because many children may self‐soothe after waking. While we excluded the number of night awakenings from our final model, and our findings on night‐time sleep duration are consistent with the existing literature, our measures of night‐time sleep may not be fully accurate. Another limitation is the absence of other crucial sleep variables, such as sleep efficiency, which have been significantly linked to various aspects of development. These variables, typically assessed through objective measures, serve as important indicators of overall sleep quality. We emphasize the need for future replications of our study incorporating objective sleep measures to provide a more comprehensive understanding of the interplay between sleep, motor, and language domains.

As mentioned, our sample was relatively small (especially the 7‐month‐olds) and was based exclusively in the UK, with participants coming from a homogeneous socio‐demographic background. Additionally, participants were mainly recruited from parent communities online, which may have further increased the selectivity of our sample. As such, our findings may not be readily generalizable to children in different geographical or demographic contexts. Motor and linguistic development unfold differently across populations, shaped by environmental, cultural, and socioeconomic factors (Adolph and Hoch 2019; Singh et al. 2024). Furthermore, variations in sleep habits across different communities and cultures could also affect the generalizability of our conclusions (Owens 2004). It would be useful for future studies to investigate the relationships between sleep regulation, motor and vocabulary development across larger samples with different sociodemographic backgrounds.

Linked to this, there is no consensus on when to consider a motor skill to have emerged (Berger and Moore 2021); accordingly, cross‐study comparisons must be made with caution. In addition, although motor milestones seem to propel linguistic development in typically developing children, alternative paths to the acquisition of language are also possible, as evidenced by the fact that children unable to walk do generally develop language. These children may gain relevant input from their environment (supporting their linguistic development) through means other than self‐produced locomotion. Reaching motor milestones is thus neither sufficient nor necessary for language acquisition (Iverson 2010). It is also evident that children demonstrate some linguistic abilities before they can sit or walk. Therefore, more studies on the associations between motor skill and vocabulary development should be run with children with limited self‐locomotion abilities, to more comprehensively understand how motor development may influence language learning.

Our measure of productive vocabulary was solely quantitative, lacking information on the complexity of the words produced, which could provide insights into patterns of vocabulary growth. Additionally, we did not capture the onset of babble, which represents the first form of complex vocal practice and serves as the foundation for word production (Vihman et al. 2009; McCune and Vihman 2001). In our additional analysis, although we did observe a positive correlation between overall motor score and productive vocabulary, we also found positive correlations between motor score and receptive vocabulary at multiple ages. Therefore, our findings on whether the motor component of vocal production impacts sleep patterns similarly to self‐locomotion remain inconclusive. Finer‐grained information on babble onset and the onset of word production could clarify the potential association between motor learning for speech sounds and sleep disturbances: Intriguingly, those disturbances observed at the onset of crawling occur almost simultaneously with the onset of babble. Considering the observed entrainment of rhythmic limb and torso movements with babble during infancy (Iverson and Thelen 1999), this point warrants further investigation.

## 5 Conclusions

The findings from our exploratory study suggest two key points. First, associations between sleep, motor skill and vocabulary may be most pronounced during periods of significant developmental change in the domains investigated, which presumably affect several aspects of a child's life (e.g., the level of linguistic input, its emotional saliency, ways in which to interact with objects and their functions, the ability to self‐regulate, practice in articulating more complex speech sounds or sequences, and so on). Second, the strength of these associations may correlate with the degree of stability of these domains at the observed age(s), with behaviors that are just beginning to emerge being more strongly associated with each other. This conclusion is in accord with the principles of Dynamic Systems Theory, which proposes that development originates from changes in systems dynamics influenced by periods of instability in behavior (Smith and Thelen 2003). In that framework, the following scenario could be theorized to link these domains in our study, based on the idea that development is self‐regulated and emerges through the interaction of multiple systems with one another and with the environment (Thelen and Smith 1994).

As infants begin to crawl, increased social interactions affect the amount and kind of speech directed to the infant (Campos et al. 2000). At the same time, infants' self‐regulatory abilities develop and gradually enable them to sustain longer periods of sleep. With the proportion of overnight sleep increasing, sleep pressure accumulates more slowly and napping decreases; learning starts to rely more and more on the alternating sleep cycles of overnight sleep (Werchan, Ji‐Soo, and Gómez 2021). This, together with the development of the brain and memory systems through sleep regulation (Ednick et al. 2009; Mason, Lokhandwala, et al. 2021), supports the consolidation and reorganization of infants' growing lexical networks and motor abilities. At this point, overnight sleep consolidation may start to have a stronger effect on their vocabulary.

Concurrently, the development of gross and fine motor skills is ongoing and opens new ways to explore the environment as well as to produce sounds. Specifically, gross motor skills support the transition to walking, and fine motor skills support vocal exploration, babbling and articulatory development, providing the basis for first word production (Vihman 2022). In turn, the transition to walking opens up opportunities for richer social interactions and environmental explorations. Children walking independently interact with caregivers for longer periods, vocalize more (Clearfield 2011) and can carry objects, which are likely to be labeled by their caregivers (Karasik, Tamis‐Lemonda, and Adolph 2014). More vocal practice and more frequent interactions facilitate productive vocabulary development (Clearfield 2011). Therefore, walking frequency starts to have a stronger effect on vocabulary.

This scenario is hypothetical and the causal relationships between sleep regulation, motor development and vocabulary growth are yet to be disentangled. This is especially the case because infant sleeping habits and motor skill development are closely associated with other factors, such as maternal and infant stress (e.g., Sadeh, Tikotzky, and Scher 2010), and parental practices that vary significantly across different geographical and sociodemographic backgrounds (Holden et al. 2022). Most existing evidence, including ours, is correlational and based on Western, Educated, Industrialized, Rich and Democratic (WEIRD) populations. Nevertheless, our findings suggest the interconnectedness of these domains and, building on the existing literature, make a case for pursuing the study of development as a dynamic process where self‐regulatory mechanisms, sleep regulation, social interactions, and motor development co‐participate in the process of vocabulary growth.

## Author Contributions

**Margherita Belia:** conceptualization, data curation, formal analysis, funding acquisition, investigation, methodology, project administration, resources, visualization, writing–original draft, writing–review and editing. **Tamar Keren‐Portnoy:** methodology, resources, supervision, writing–review and editing. **Marilyn Vihman:** resources, supervision, writing–review and editing.

## Supporting information

Supporting Information S1

## Data Availability

The data that support the findings of this study are available on the OSF database (link: https://osf.io/es83q/; DOI: https://doi.org/10.17605/OSF.IO/ES83Q).
