# Supplementary material for: Exploring Developmental Connections: Sleep Patterns, Self‐Locomotion, and Vocabulary Growth in Early Childhood
Source: Infancy. 2025 Jan 24;30(1):e12650. doi: 10.1111/infa.12650 (PMC11760629; doi:10.1111/infa.12650)
Supplement: Supplementary file 1 — Supporting Information S1 [file INFA-30-0-s001.docx]

1

2

3

4

5

6 Supplementary Materials

7

8

9 Appendix A. Model assumptions

10

11

12

13


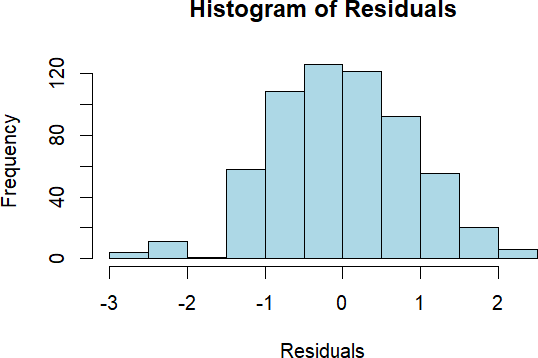
14

15

16

For Peer

17

18

19

20

21

22

23

24

25

26

27

Review Only

28

29

30 *Figure S1. Histogram of residuals for Model 1.*

31

32


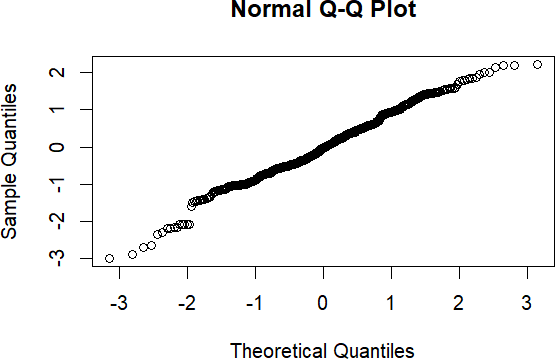
33

34

35

36

37

38

39

40

41

42

43

44

45

46

47

48

49 *Figure S2. QQ plot for Model 1.*

51

52

53

54

55

56

57

58

59

60

1

2

3


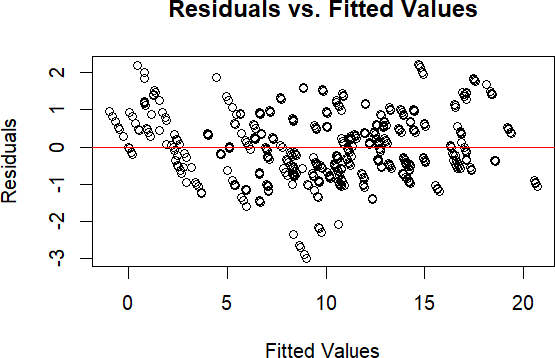
4

5

6

7

8

9

10

11

12

13

14

15

16

For

17

18

19

20

21 *Figure S3. Residuals vs. Fitted values plot for Model 1.*

Peer

22

23


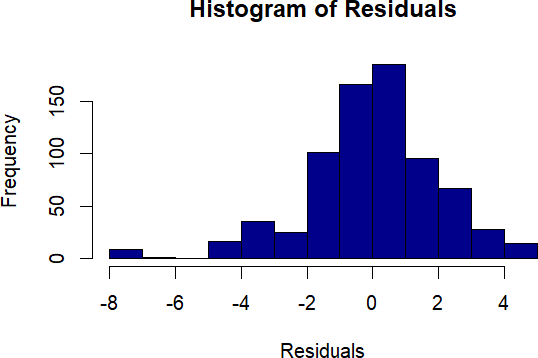
24

25

26

27

Review Only

28

29

30

31

32

33

34

35

36

37

38

39

40

41

42 *Figure S4. Histogram of residuals for Model 2.*

43

44

45

46

47

48

49

50

51

52

53

54

55

56

57

58

59

60

1

2

3

4


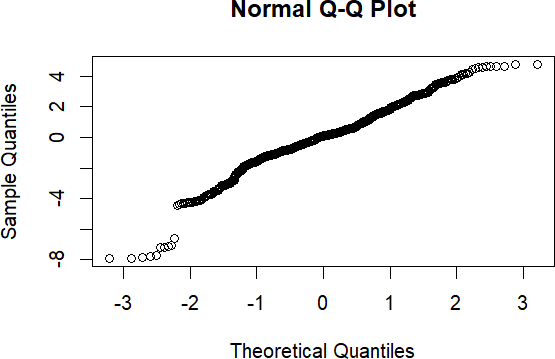
5

6

7

8

9

10

11

12

13

14

15

16

For

17

18

19

20

Peer Review

21

22 *Figure S5. QQ plot for Model 2.*

23

24


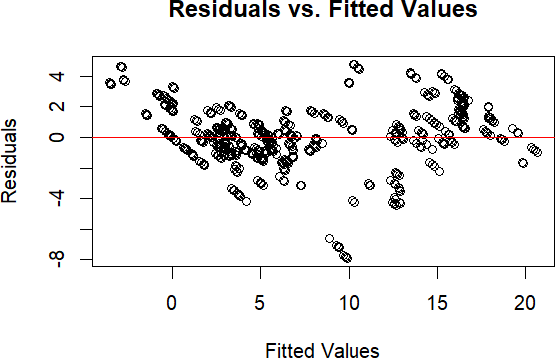
25

26

27

28

29

30

31

32

33

34

35

36

37

38

Only

39

40

41

42

43 *Figure S6. "Residual vs. Fitted values plot for Model 2.*

44

45

46

47

48

49

50

51

52

53

54

55

56

57

58

59

60
